# Tetrahedral framework nucleic acids carrying tranexamic acid to alleviate ultraviolet B‐induced skin pigmentation

**DOI:** 10.1016/j.mtbio.2026.103287

**Published:** 2026-05-27

**Authors:** Tao Cai, Junyang Huang, Li Chen, Yi Lin, Kaiqun Liu, Yuanyuan Deng, Jiajin Feng, You Wang, Xiaoyan Ding

**Affiliations:** a School of Medicine, University of Electronic Science and Technology of China, Chengdu, 610054, China; b Sichuan Eye Medical Center, Sichuan Provincial People's Hospital, University of Electronic Science and Technology of China, Chengdu, 610072, China; c The First School of Clinical Medicine, Southern Medical University, Guangzhou, 510515, China; d Department of Ophthalmology, General Hospital of Central Theater Command, Wuhan, 430070, China; e Chengdu Yun Hai Tetrahedron Biotech Co. Ltd., Chengdu, 610036, China

**Keywords:** Skin damage, tFNAs, Nanoparticle, TXA

## Abstract

Hyperpigmentation disorders such as melasma require topical therapies that are both effective and well tolerated, yet many current depigmenting agents are limited by irritation or inconsistent efficacy. Tranexamic acid (TXA) is a clinically used small molecule with emerging anti-pigmentation utility, but its topical performance is often constrained by insufficient penetration and limited cellular access. In this work, we engineered a tetrahedral framework nucleic acid (tFNA)–enabled TXA formulation (tFNAs–TXA) to enhance topical delivery and anti-melanogenic activity. tFNAs–TXA was successfully constructed with preserved nanostructure, physicochemical stability, and efficient cellular internalization. Functionally, tFNAs–TXA significantly attenuated α-MSH–induced melanogenesis in B16 cells without overt cytotoxicity and downregulated the cAMP–p-CREB–MITF–TYR signaling cascade at both protein and mRNA levels. In a UVB-induced mouse model, topical tFNAs–TXA markedly reduced dorsal pigmentation and mitigated UVB-associated histopathological changes, including melanin deposition, collagen disorganization, and epidermal hyperplasia, achieving depigmenting efficacy with better gross tolerability. Together, these findings establish tFNAs–TXA as a concise, non-invasive delivery strategy that potentiates TXA for safer and more effective management of UV-related hyperpigmentation.

## Highlights

• Programmable tFNA nanocarrier enhances TXA transdermal delivery.
• Achieves HQ-comparable depigmentation with improved skin tolerability.
• TFNAs-TXA inhibits melanogenesis by silencing the cAMP-p-CREB-MITF-TYR axis.

## Graphical abstract

**Figure:**
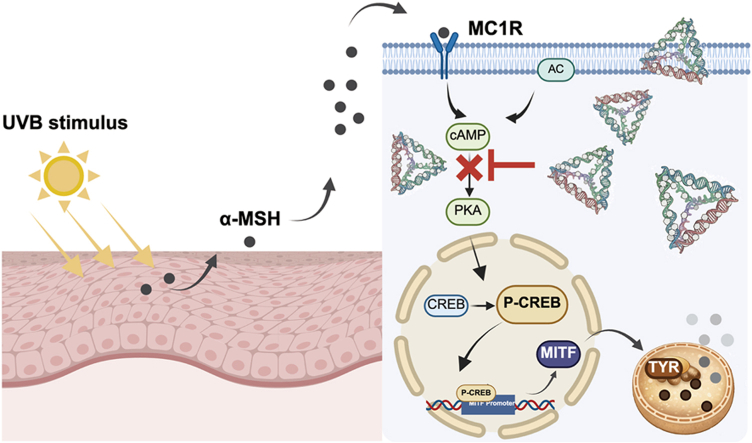

## 1 Introduction

Hyperpigmentary disorders, particularly melasma [1], freckles [2], senile plaques, and postinflammatory hyperpigmentation (PIH) [3], are among the most prevalent and clinically challenging pigmentary conditions [4]. They often exert a disproportionate psychosocial impact and remain difficult to manage in routine practice. Fundamentally, these conditions are driven by the overproduction and aberrant distribution of melanin. Melanin is synthesized by melanocytes located in the basal epidermis [5]. Melanosomes are specialized organelles responsible for melanin biogenesis and storage within melanocytes [6]. As melanosomes mature, they are transferred to keratinocytes and distributed throughout the epidermis, ultimately determining visible skin colour [[7], [8], [9]]. Therefore, interventions that can efficiently penetrate the skin to attenuate melanogenesis at the basal layer represent a crucial strategy for treating hyperpigmentation. Current therapeutic paradigms include topical depigmenting agents (e.g., hydroquinone and derivatives, retinoids, azelaic acid, kojic acid/arbutin) [10,11], systemic or topical anti-oxidative/anti-inflammatory regimens [12], and procedure-based interventions such as chemical peeling and energy-based devices [13]. Nevertheless, clinical outcomes are frequently inconsistent, and treatment-limiting adverse effects are common. Barrier impairment, and inflammation-driven rebound pigmentation can compromise efficacy and tolerability [14], while procedural modalities are operator- and parameter-dependent and may precipitate PIH in predisposed individuals [15]. Therefore, there is a clear unmet need for strategies that deliver reliable anti-hyperpigmentation efficacy with improved cutaneous delivery and a favorable safety profile.

Tranexamic acid (TXA) has gained substantial attention as a therapeutic option for melasma and related hyperpigmentation. TXA is generally considered to modulate fibrinolysis-associated pathways and downstream inflammatory and vascular mediators, thereby attenuating melanogenesis and pigment transfer [[16], [17], [18]]. Although clinical and experimental evidence supports its depigmenting potential, the therapeutic performance of TXA is often limited by formulation and delivery constraints. Oral TXA, while effective in selected patients, requires careful safety evaluation and may be restricted by patient acceptance and contraindications [19,20]. Topical TXA is attractive from a safety standpoint but is frequently hindered by insufficient transdermal penetration and suboptimal local bioavailability within the relevant epidermal and dermal compartments, leading to limited efficacy [[21], [22], [23]]. Accordingly, improving the cutaneous delivery of TXA-while preserving or enhancing safety-represents a key translational barrier and a priority for the development of next-generation anti-hyperpigmentation interventions.

Advanced functional nanomaterials have emerged as a cornerstone of modern biomedicine, offering transformative potential across a broad spectrum of applications ranging from high-sensitivity disease detection to precision therapeutic interventions [[24], [25], [26]]. Within this versatile landscape, tetrahedral framework nucleic acids (tFNAs) are programmable DNA nanostructures self-assembled through complementary base pairing [27]. tFNAs exhibit well-defined geometry, favorable biocompatibility, structural stability, and relatively efficient cellular internalization, supporting their development as delivery platforms for small molecules and nucleic acid cargos [[28], [29], [30], [31]]. Prior studies have established the canonical architecture and nanoscale morphology of tFNAs and demonstrated their feasibility in enhancing cellular uptake and biological activity [30]. Crucially, following cellular internalization, these DNA-based nanocarriers undergo natural degradation by endogenous nucleases into non-toxic nucleotides, thereby ensuring excellent biosafety and facilitating the dynamic release of loaded cargos [32]. Based on these properties, we hypothesized that incorporating TXA into a tFNA-based carrier could improve transdermal transport and cellular access of TXA, thereby strengthening anti-hyperpigmentation efficacy while minimizing irritancy and systemic exposure.

In this study, we engineered and characterized a tFNA-carried TXA system (tFNAs–TXA) and focused on three translationally relevant objectives: (i) improving anti-hyperpigmentation efficacy, (ii) enabling or enhancing transdermal delivery, and (iii) ensuring an acceptable safety profile. We systematically evaluated the physicochemical properties and cellular uptake of tFNAs–TXA and investigated its performance in pigmentation-related experimental models. By addressing the central limitation of conventional topical TXA-insufficient penetration and limited access to target cells—this work seeks to establish a safer and more effective transdermal TXA delivery strategy for hyperpigmentation therapy.

## 2 Results and discussion

### 2.1 Synthesis and characterization of tFNAs-TXA

**Fig. 1:**
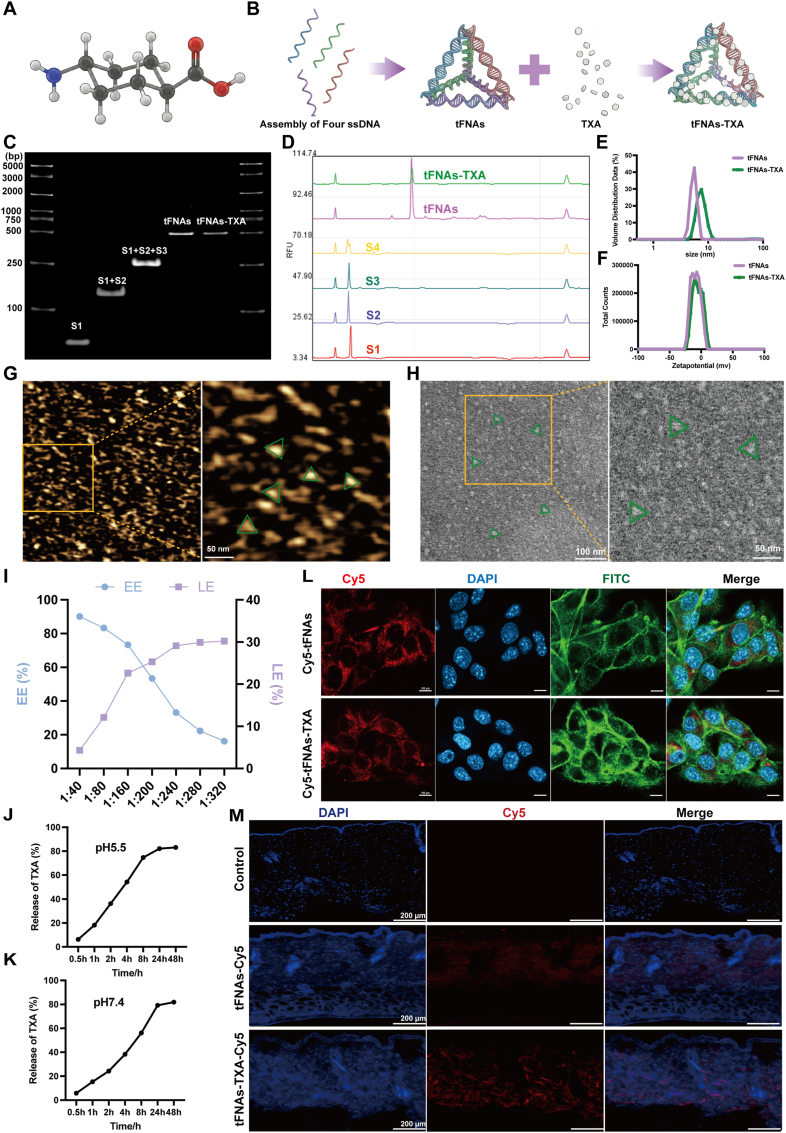
**Design, synthesis, and characterization of tFNAs-TXA.** (A) The molecular structure of TXA. (B) Schematic diagram of the synthesis process of tFNAs and tFNAs-TXA. (C) Polyacrylamide gel electrophoresis (PAGE) confirmed the successful synthesis of tFNAs and tFNAs-TXA. (D) High-performance capillary electrophoresis (HPCE) verified the successful assembly of tFNAs and their conjugation with TXA. (E) The larger size of tFNAs-TXA than tFNAs was detected by dynamic light scattering (DLS). (F) The zeta potential of tFNAs and tFNAs-TXA was determined by DLS. (G) The AFM image of tFNAs-TXA. (H) The TEM image of tFNAs-TXA. (I) Encapsulation efficiency (EE) and loading efficiency (LE) of tFNAs-TXA at varying molar feed ratios. (J, K) In vitro cumulative release profiles of TXA from the tFNAs-TXA complexes under (J) mildly acidic (pH 5.5) and (K) physiological (pH 7.4) conditions. (L) Representative confocal images showing the cellular uptake of Cy5-labeled tFNAs and tFNAs-TXA at 16 h (red: Cy5-labeled tFNAs and tFNAs-TXA, blue: nucleus, green: cytoskeleton). (M) Representative confocal fluorescence images of ex vivo skin cryosections demonstrating the transdermal penetration of the Cy5-labeled nanostructures (blue: DAPI-stained nuclei, red: Cy5-labeled nanostructures).

To construct the delivery system, TXA (chemical structure shown in Fig. 1A) was loaded onto the assembled tFNAs following the synthetic route outlined in Fig. 1B. Briefly, tetrahedral framework nucleic acids (tFNAs) were successfully synthesized using four equimolar single-stranded DNAs, as outlined in Methods. Each single-stranded DNA was designed to contain three complementary domains that, through specific programmed complementary base pairing, formed a triangular facet and hybridized with the other three ssDNAs, yielding a rigid tetrahedral nanostructure. After assembly, purified tFNAs were incubated with TXA at 4 °C under gentle oscillation for 6 h to obtain tFNAs-TXA.

Successful formation of tFNAs and tFNAs-TXA was first verified by 8% polyacrylamide gel electrophoresis (PAGE). Compared with individual ssDNAs, assembled tFNAs exhibited a distinct mobility shift with retarded migration, consistent with the theoretical construct of a tetrahedron composed of four ssDNA strands and in agreement with previous reports (Fig. 1C) [33]. High-performance capillary electrophoresis (HPCE) further confirmed the successful generation of tFNAs and tFNAs-TXA (Fig. 1D), yielding profiles consistent with the PAGE results. Dynamic light scattering (DLS) analysis showed the particle sizes of 5.61 nm for tFNAs and 7.53 nm for tFNAs-TXA, respectively by Fig. 1E, indicating that TXA loading did not substantially altered the overall nanostructure size. Subsequently, Zeta potential measurements demonstrated that tFNAs carried a surface charge of −6.99 mV, while tFNAs-TXA, exhibited a zeta potential of −11.96 mV (Fig. 1F), confirmed colloidal stability following TXA loading. Mechanistically, the stable loading of TXA onto tFNAs is synergistically driven by electrostatic interactions (between the TXA amino group and DNA phosphate backbone), spatial groove entrapment, and hydrogen bonding. This non-covalent association is supported by the observed decrease in zeta potential and the slight increase in particle size, which occur without disrupting the structural integrity of the DNA framework. The characteristics of tFNAs were further studied using atomic force microscopy (AFM)(Fig. 1G) and transmission electron microscopy (TEM), the results showed a triangular morphology for the successfully synthesized tFNAs-TXA, similar to previous findings (Fig. 1H) [34,35]. To determine the optimal formulation and precise loading stoichiometry, the encapsulation efficiency (EE) and loading efficiency (LE) were quantified across a gradient of feed ratios (Fig. 1I). At the optimal 1:160 M ratio, the system achieved an excellent thermodynamic balance with a high EE (∼73%) and a substantial LE (>20%), effectively carrying approximately 117 TXA molecules per tFNA nanostructure. Furthermore, the in vitro release profile of the optimized tFNAs-TXA complex was investigated to evaluate its stability and responsiveness. As shown in Fig. 1J and K, the complex exhibited a slow and sustained release of TXA under normal physiological conditions (pH 7.4), confirming excellent colloidal stability and minimal premature drug leakage. In contrast, under mildly acidic conditions (pH 5.5, mimicking the intracellular endo/lysosomal microenvironment), a significantly accelerated and responsive release pattern was observed, which facilitates efficient drug unloading upon cellular internalization. To investigate the ability and efficiency of tFNAs and tFNAs-TXA to enter B16 cells, confocal microscopy was used to detect the uptake of Cy5-labeled tFNA and tFNAs-TXA. After 16 h, Cy5-labeled tFNA and tFNAs-TXA were widely distributed in the cytoplasm, indicating favorable cellular uptake (Fig. 1L). Crucially, to directly validate the transdermal delivery efficiency of the system, ex vivo skin penetration experiments were conducted. Confocal fluorescence imaging of skin cryosections (Fig. 1M) revealed that the Cy5-labeled tFNAs-TXA complexes efficiently traversed the stratum corneum barrier and penetrated deep into the epidermis and basal layers, where melanocytes reside. Collectively, these results confirm the successful construction and structural integrity of tFNAs–TXA and demonstrate its physicochemical stability and precise drug-loading capacity, pH-responsive release profile, efficient cellular internalization. From a translational perspective, the nanoscale size, negative surface potential, and robust uptake profile support the feasibility of using tFNAs as a topical delivery platform to enhance intracellular access of TXA - an important prerequisite for improving local efficacy while maintaining an acceptable safety profile. Subsequent experiments are required to quantify transdermal transport, therapeutic performance against melanogenesis, and biocompatibility under clinically relevant exposure conditions.

### 2.2 tFNAs-TXA inhibits α-MSH-induced pigmentation in vitro

**Fig. 2:**
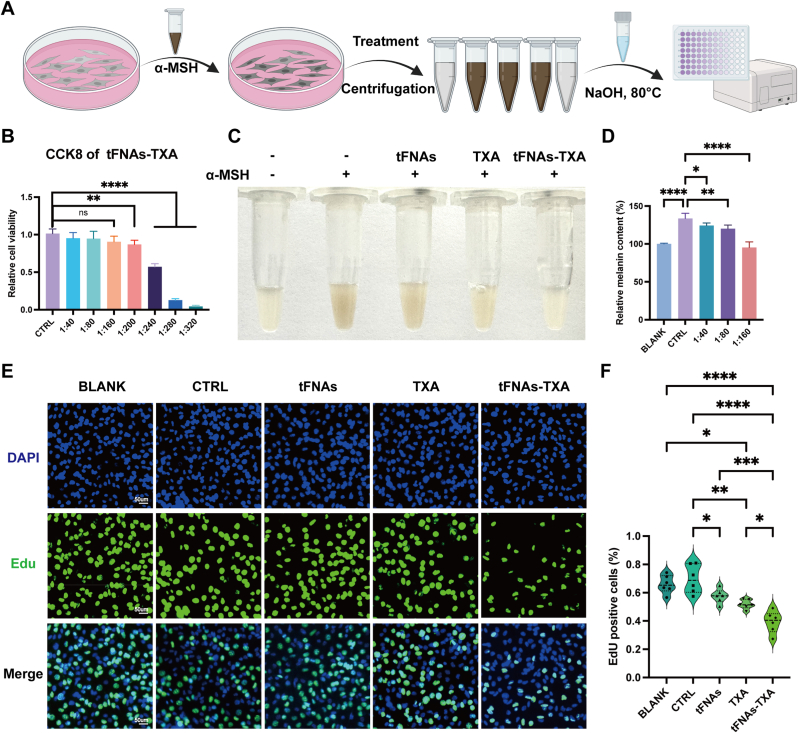
**Assessment of the melanogenesis-inhibitory effects of tFNAs-TXA** (A) Schematic workflow of the melanin inhibition assay. (B, C) CCK-8 assays showing the (B) cytotoxicity and (C) optimal concentration screening of tFNAs-TXA in B16 cells. (D) Morphological changes of B16 cells under different treatments. (E, F) EdU proliferation assay. Representative fluorescence images (E) and statistical analysis of EdU-positive rates (F) across the indicated groups (n = 6 independent biological replicates). Data are presented as mean ± SD. Statistical significance was assessed by one-way ANOVA test followed by Tukey's post-hoc test; *p < 0.05, **p < 0.01, ***p < 0.001, and ****p < 0.0001.

Prior to investigating the anti-melanogenic efficacy, we first evaluated the biological safety of the tFNAs–TXA complexes in B16 cells using a CCK-8 assay. Cells were exposed to tFNAs–TXA prepared at different tFNA:TXA molar ratios (1:40 to 1:320). As shown in Fig. 2B, no significant reduction in cell viability was observed at ratios of 1:40, 1:80, or 1:160 compared with the control group, indicating acceptable cytocompatibility within this range.

Next, an in vitro pigmentation model was established by stimulating B16 cells with alpha-melanocyte stimulating hormone (α-MSH) (Fig. 2A). α-MSH markedly increased intracellular melanin content in the model group, confirming successful induction of melanogenesis. Compared with the α-MSH treated control, high-dose tFNAs-TXA (1:160) significantly reduced melanin accumulation (Fig. 2C and D). Based on the balance between cytocompatibility and anti-melanogenic efficacy, the formulation corresponding to tFNAs 250 nM and TXA 40 μM (tFNA:TXA = 1:160) was selected for subsequent in vitro and in vivo studies.

To further examine the biological impact of tFNAs–TXA, cell proliferation was assessed using EdU incorporation. tFNAs–TXA significantly decreased EdU-positive cells relative to the control group, whereas tFNAs alone or TXA alone produced only modest effects (Fig. 2E and F). These findings suggest that the combined formulation more effectively restrains α-MSH–associated cellular activation than either component administered separately.

### 2.3 tFNAs-TXA suppresses UVB-induced pigmentation and skin damage in vivo

**Fig. 3:**
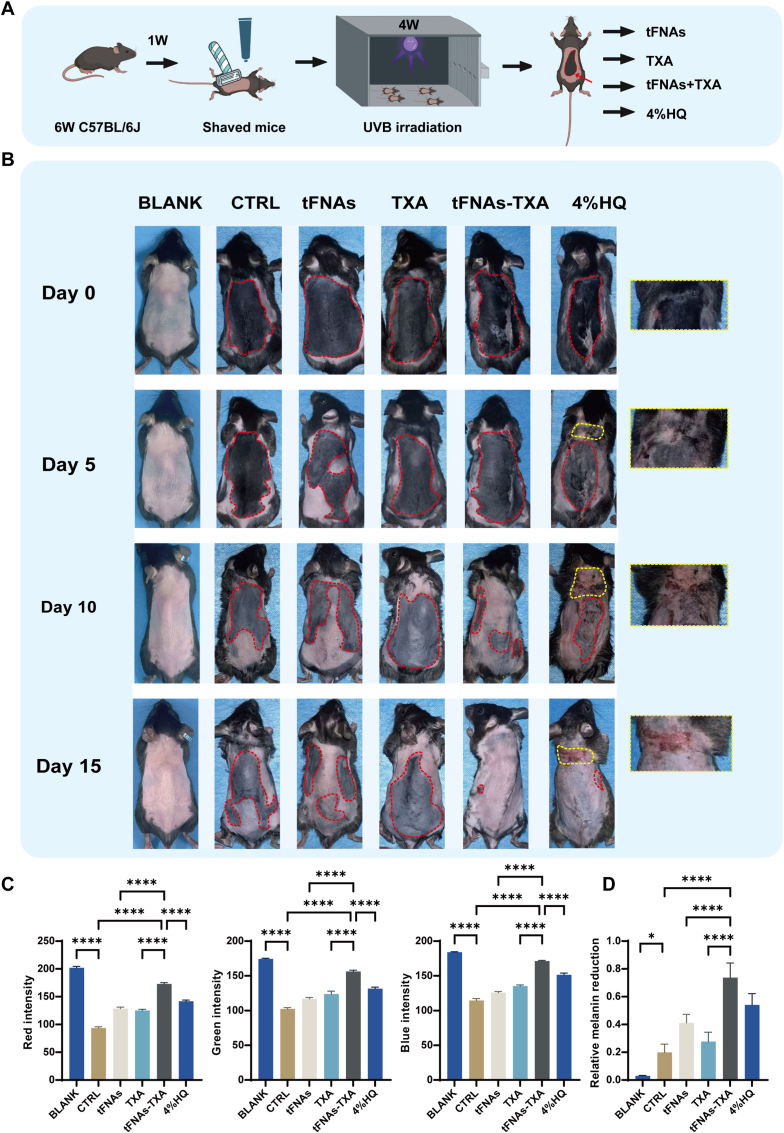
**Evaluation of the therapeutic efficacy of tFNAs–TXA in a UV-induced hyperpigmentation mouse model.** (A) Schematic description of the establishment of the mouse ultraviolet irradiation model and the design of the animal experiments. (B) Pictures of black spots on the back skin of the mice at days 0, 5, 10,and 15 (before treatment and on day 5, 10, and 15 after treatment). Red dotted line indicates the area with melanin deposition across different time points within the same mice. Yellow dotted line represents the damaged area of the skin after hydroquinone treatment. (C) RGB channel separation–based quantitative analysis of pigment signals in the red, green, and blue channels using ImageJ. (D) The relative reduction of melanin on the backs of mice after ultraviolet irradiation and drug treatment. Statistical significance was assessed by one-way ANOVA test followed by Tukey's post-hoc test (n = 6 per group; *p < 0.05, **p < 0.01, ***p < 0.001, and ****p < 0.0001).

Melasma and other acquired hyperpigmentation disorders are commonly models in vivo to enable quantitative evaluation of topical anti-pigmentation interventions [36]. Here, we established a UVB-induced pigmentation model in C57BL/6J mice (Fig. 3A). The dorsal skin was selected because it provides a relatively large and flat surface that allows standardized ultraviolet B exposure, convenient topical application, and longitudinal photographic documentation. Moreover, it is widely used in melanin-related preclinical studies [37]. After model induction, mice were randomized into five groups (blank, Ctrl, tFNAs, TXA, and tFNAs–TXA). Based on the in vitro screening results, the optimal concentration (250 nM tFNAs and 40 μM TXA) was translated to the topical formulations for the subsequent in vivo evaluation. Representative gross images of dorsal pigmentation at days 0, 5, 10, and 15 are shown in Fig. 3B. The red dotted outline indicates the region of melanin deposition tracked across time points within the same mouse, enabling consistent within-animal comparison. 4% hydroquinone (HQ) was used as a positive control, as it is a clinically established depigmenting agent that inhibits tyrosinase activity and is widely used in topical formulations for hyperpigmentation treatment [10]. As expected, while effective, it showed visible treatment-associated skin damage (yellow dotted outline, Fig. 3B), consistent with the well-recognized irritancy potential of high-concentration HQ.

To quantify pigmentation, we performed ImageJ-based RGB channel separation and signal analysis (Fig. 3C). Compared with the Ctrl group, topical tFNAs–TXA significantly reduced pigmentation signals across the RGB channels, consistent with the representative images (Fig. 3B and C). The relative reduction of melanin signal further confirmed that tFNAs–TXA produced a robust depigmenting effect (n = 6; Fig. 3D; significance as indicated). Importantly, the magnitude of improvement with tFNAs-TXA was comparable to that achieved with the commercial tyrosinase inhibitor 4% HQ (Fig. 3D), while avoiding the grossly visible skin injury observed after HQ treatment in the model (Fig. 3B). In contrast, free TXA did not produce a significant reduction in pigmentation compared with Ctrl (Fig. 3D), consistent with limited transdermal delivery and insufficient local bioavailability when applied topically. tFNAs alone showed minimal or no depigmenting effect in vivo, supporting the rationale that the therapeutic benefit arises from the carrier–enabled delivery of TXA rather than from the carrier itself.

Collectively, these in vivo findings demonstrate that loading TXA onto tFNAs substantially enhances the topical anti-pigmentation efficacy of TXA in a UV-induced hyperpigmentation model, reaching an effect comparable to 4% HQ with better gross tolerability. Further histological and safety assessments are presented in the following sections.

### 2.4 tFNAs–TXA mitigates UVB-induced histopathological alterations in murine skin

**Fig. 4:**
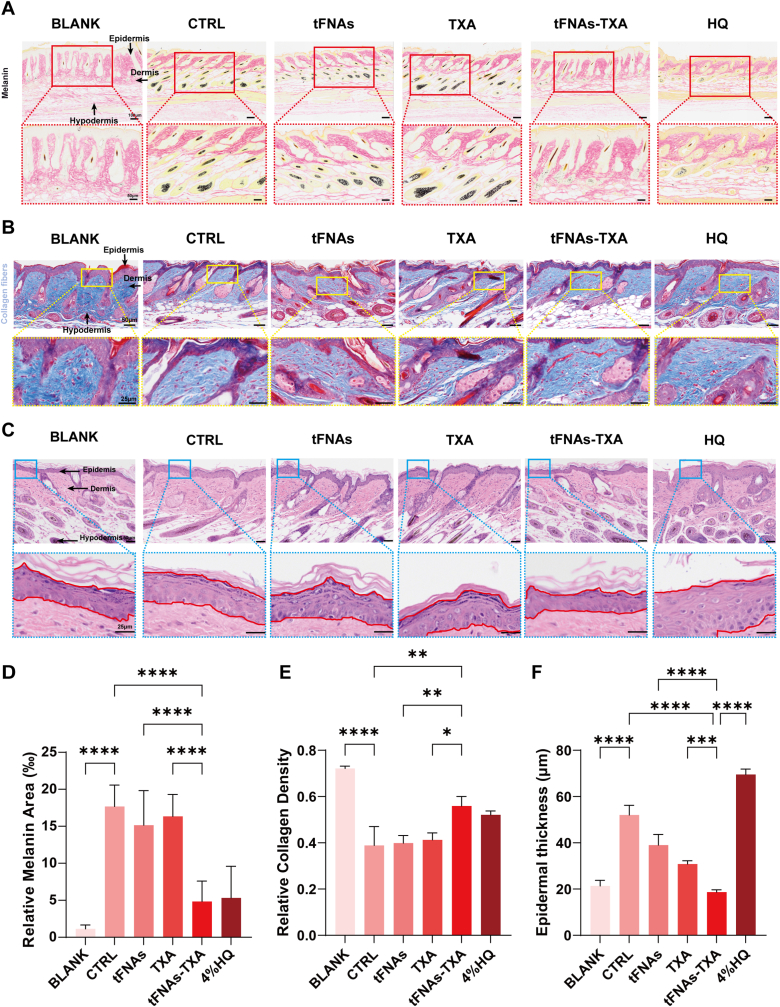
**Histological analysis of anti-pigmentation effects. (A)** Melanin was stained black by Masson-Fontana silver staining. (B) Masson's trichrome staining showing collagen fibers (blue) in the dermis. (C) H&E staining detects epidermal layer thickness to assess skin inflammation. Statistical analysis of melanin content (D), collagen density (E), and epidermal thickness (F). The data are mean ± SD (n = 6, *p < 0.05, **p < 0.01, ***p < 0.001, and ****p < 0.0001, determined by one-way ANOVA followed by Tukey's post-hoc test).

To further determine whether the macroscopic depigmenting effect of tFNAs-TXA was accompanied by improvements in tissue-level injury and architecture, we performed histopathological and special staining analyses on dorsal skin sections from UVB-irradiated mice. Masson–Fontana silver staining revealed a marked reduction in melanin deposition in skin tissues treated with tFNAs-TXA (Fig. 4A). Quantitative analysis confirmed that the melanin-positive area was significantly lower in the tFNAs-TXA group than in the CTRL, TXA, and tFNAs groups (all p < 0.0001, Fig. 4D), and was comparable to that observed in the 4% HQ group (p > 0.05).

Because UVB exposure can disrupt dermal extracellular matrix organization [38,39], we next evaluated collagen fiber distribution using Masson's trichrome staining, in which collagen fibers appear blue. As shown in Fig. 4B, the CTRL group exhibited sparse and disorganized collagen bundles, indicating significant damage to the skin structure. Collagen area fraction analysis demonstrated a higher dermal collagen content in the tFNAs-TXA group compared with the CTRL (p < 0.01), tFNAs (p < 0.01), and TXA groups (p < 0.05, Fig. 4E), which was comparable to the level observed in the 4% HQ group. These data suggest that tFNAs-TXA confers a significant protective and restorative effect against radiation-induced collagen fiber damage.

Inflammation after UVB injury is commonly accompanied by epidermal hyperplasia, and epidermal thickening is frequently used as a histological indicator of irritation and inflammatory remodeling [40,41]. Based on hematoxylin and eosin (H&E) staining, the epidermal and dermal structures of Blank group remain intact (Fig. 4C). However, the UVB treated control group showed a significant increase in epidermal thickness (p < 0.0001, Fig. 4F). Treatment with tFNAs-TXA attenuated UVB-induced epidermal hyperplasia, with the epidermal thickness significantly lower than that in the CTRL (p < 0.0001), tFNAs (p < 0.0001), TXA (p < 0.01) and 4% HQ group(p < 0.0001). Notably, epidermal thickness in the 4% HQ group increased significantly compared to the CTRL group (p < 0.001), indicating that tFNAs-TXA effectively suppresses UVB-induced epidermal hyperplasia and provides superior protection of epidermal architecture compared with TXA and 4% HQ.

Collectively, these results demonstrate that tFNAs–TXA not only effectively alleviates UV-induced hyperpigmentation, but also preserves dermal collagen organization and maintains epidermal structural integrity. Compared with 4% HQ, tFNAs-TXA exhibits a favorable balance between anti-pigmentation and skin tissue protection.

### 2.5 Elucidation of the core therapeutic mechanism via multi-dimensional bioinformatics analysis

**Fig. 5:**
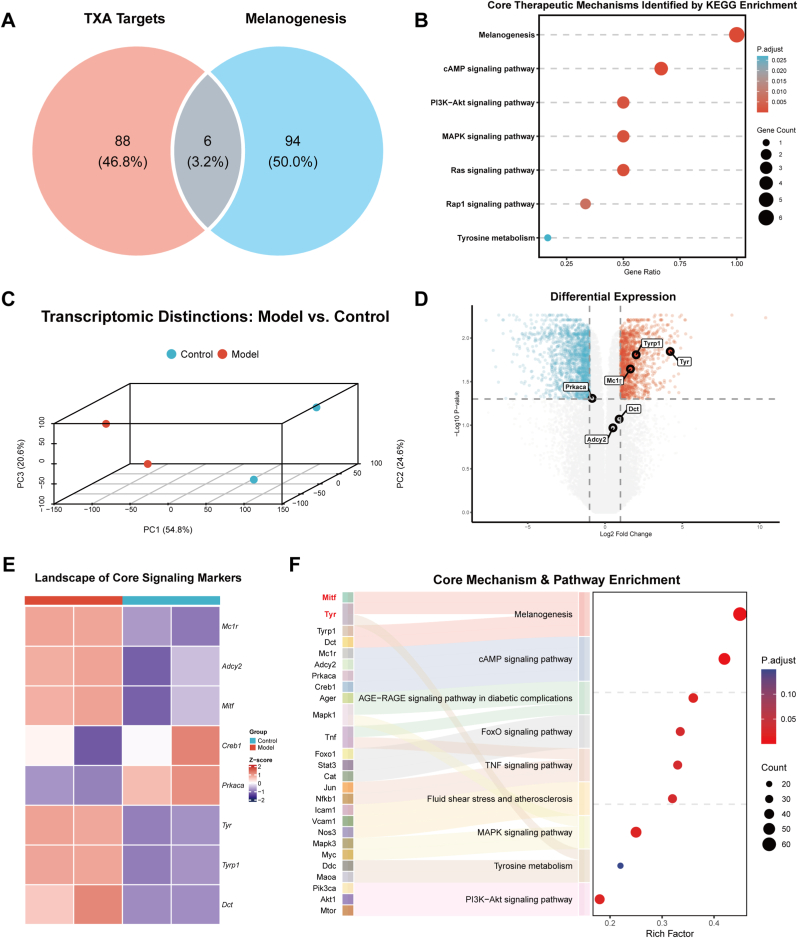
**Bioinformatics analysis identifying the core therapeutic mechanism of tFNAs-TXA.** (A) Venn diagram showing the intersection between predicted targets of TXA and melanogenesis-related genes. (B) KEGG pathway enrichment analysis of the common targets. (C) 3D Principal Component Analysis of the GSE54359 transcriptomic dataset. (D) Volcano plot showing differentially expressed genes between Model and Control groups (red: upregulation, blue: downregulation). (E) Heatmap of the expression levels of core signaling markers across samples. (F) Integrated Sankey-Bubble plot visualizing the gene-pathway flow and enrichment significance.

To systematically decode the molecular mechanism driving the depigmentation activity of tFNAs-TXA, we employed an integrated strategy combining pharmacological target prediction with transcriptomic profiling [42,43]. First, the predicted pharmacological targets of TXA were intersected with established melanogenesis-associated genes, identifying six high-confidence core targets (Fig. 5A). KEGG pathway enrichment analysis [44] revealed a remarkable functional convergence, with these targets predominantly enriched in the cAMP signaling pathway and Melanogenesis (Fig. 5B). This bioinformatics evidence posits that tFNAs-TXA likely exerts its therapeutic effects by specifically modulating this signaling cascade.

To corroborate these findings within the transcriptional landscape of the disease state, we analyzed the GSE54359 dataset. Principal Component Analysis (PCA) confirmed distinct transcriptomic profiles between the Model and Control groups (Fig. 5C). Focusing on the predicted cAMP-melanogenesis axis, both the volcano plot (Fig. 5D) and heatmap (Fig. 5E) demonstrated a significant upregulation of key pathway regulators in the Model group. To visualize the regulatory logic, we constructed an integrated Sankey-Bubble plot (Fig. 5F). This visualization elucidates a hierarchical signal transduction cascade: Creb1 emerged as a key upstream regulator, linking the cAMP signaling pathway to the central hub genes Mitf and Tyr. Collectively, these multi-dimensional analyses suggest that hyperactivation of the cAMP-CREB-MITF-TYR axis is a critical pathological feature, and provide a compelling rationale that tFNAs-TXA inhibits melanogenesis by suppressing this specific axis.

### 2.6 tFNAs–TXA inhibits melanogenesis through suppression of the cAMP–p-CREB–MITF–TYR axis

**Fig. 6:**
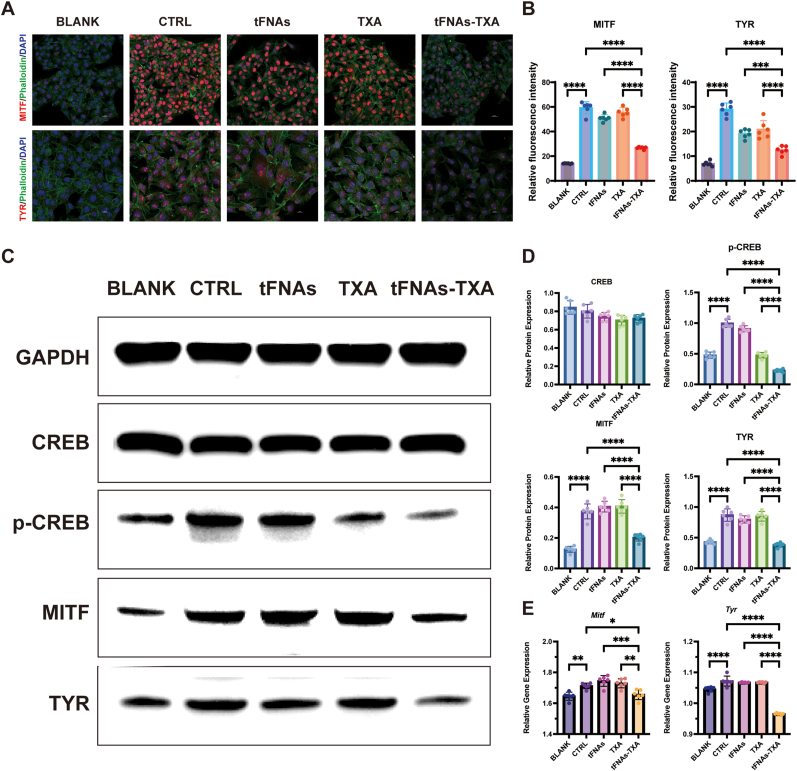
**tFNAs-TXA inhibits melanin synthesis by suppressing the cAMP/CREB/MITF signaling pathway.** (A) Representative immunofluorescence images showing the intracellular expression of MITF and TYR in B16 cells. Nuclei were stained with DAPI, cytoskeleton with Phalloidin, and target proteins with red fluorescence. (B) Quantitative analysis of relative fluorescence intensity for MITF and TYR (n = 6). (C) Representative Western blot bands showing the protein expression of CREB, p-CREB, MITF, and TYR (GAPDH served as the loading control). (D) Quantitative analysis of protein expression levels for Total CREB, p-CREB, MITF, and TYR, normalized to GAPDH. (E) Relative mRNA expression levels of *Mitf* and *Tyr* determined by RT-qPCR. Data are presented as mean ± SD (n = 6 independent biological replicates; *p < 0.05, **p < 0.01, ***p < 0.001, and ****p < 0.0001 by one-way ANOVA followed by Tukey's post-hoc test).

Although our bioinformatics analysis identified six core targets within the melanogenesis cascade, they functionally belong to the same signaling pathway. To avoid redundant validation and efficiently confirm the overall blockade of this predicted axis, we selected TYR—the ultimate rate-limiting enzyme of melanin synthesis—and its direct upstream regulators (MITF and p-CREB) for in-depth experimental validation. Accordingly, we initially investigated the expression of these pivotal markers using immunofluorescence to determine if phenotypic depigmentation was driven by specific protein-level alterations. In B16 cells stimulated with α-MSH, strong fluorescence signals were observed in the Model (CTRL) group. However, treatment with tFNAs–TXA precipitated a substantial reduction in MITF nuclear accumulation and TYR cytoplasmic immunoreactivity compared with the Control group (Fig. 6A). Quantitative analysis confirmed that the relative fluorescence intensities of both markers were significantly suppressed in the tFNAs–TXA group (Fig. 6B). These findings demonstrate that tFNAs–TXA effectively arrests the melanogenic program by downregulating its key transcriptional and enzymatic components.

To further elucidate the upstream molecular hierarchy, we analyzed the cAMP–p-CREB–MITF–TYR signaling cascade by Western blotting (Fig. 6C). Physiologically, TYR catalyzes the rate-limiting conversion of L-tyrosine to dopaquinone [[45], [46], [47]]. Under UV or hormonal stimulation, elevated intracellular cAMP triggers the phosphorylation of CREB (p-CREB), which in turn transactivates MITF to drive TYR expression [48].

Western blot analysis indicated that while total CREB levels remained invariant across all experimental groups (Fig. 6D), the levels of p-CREB, MITF, and TYR were dramatically upregulated in the Model group. Conversely, tFNAs–TXA treatment significantly reversed this upregulation (Fig. 6D). Crucially, the tFNAs-TXA complex elicited a significantly more potent suppression of p-CREB, MITF, and TYR protein levels compared to equimolar TXA monotherapy (all *p* < 0.001). This comparison strongly suggests that the tFNA nanocarrier enhances the pharmacological potency of TXA by facilitating intracellular delivery.

In alignment with the proteomic findings, RT-qPCR analysis confirmed that tFNAs-TXA effectively downregulated the mRNA transcription of both *Mitf* and *Tyr* (Fig. 6E). Previous studies have established that tFNAs possess superior cell penetration capabilities, enabling efficient cellular entry by minimizing electrostatic repulsion. Our results suggest that tFNAs-TXA leverages this enhanced internalization to coordinately inhibit the cAMP–p-CREB–MITF–TYR axis. Collectively, these data provide a molecular basis for the superior anti-pigmentation efficacy of tFNAs-TXA, which is achieved by blocking the cAMP-driven phosphorylation of CREB and consequently silencing the downstream melanogenic machinery.

## 3 Conclusions

Overall, we developed a tFNA-enabled tranexamic acid formulation (tFNAs-TXA) with preserved nanoscale structure, good stability, and efficient cellular uptake. tFNAs-TXA significantly inhibited α-MSH–induced melanogenesis in B16 cells and suppressed the cAMP–p-CREB–MITF–TYR axis. In a UVB-induced mouse model, it markedly reduced pigmentation and improved tissue architecture, achieving efficacy comparable to 4% hydroquinone with better gross tolerability. These findings support tFNAs-TXA as a practical topical strategy for melasma and PIH. In the future, it may be developed into clinically relevant dosage forms (e.g., creams/gels/patches) and used as an adjunct to procedural therapies to lower irritation-related pigmentation risk, while providing a modular platform for broader anti-pigmentation drug delivery.

## 4 Materials and Methods

### 4.1 Synthesis and characterization of tFNAs-TXA

The four component ssDNA strands (Table 1) were synthesized and purified via HPLC by Sangon Biotech (Shanghai, China). tFNAs were self-assembled by mixing equimolar amounts of these strands in TM buffer (10 mM Tris-HCl, pH 8.0; 50 mM MgCl_2_) at a final concentration of 100 μM. The reaction involved heating to 95 °C for 10 min and cooling to 4 °C for 20 min. Subsequently, Tranexamic Acid (TXA), purchased from MedChemExpress (Monmouth Junction, NJ, USA), was loaded onto the tFNAs via physical adsorption by incubating the mixture (1:160 M ratio of tFNAs to TXA) at 4 °C overnight with oscillation. Nanostructure morphology was verified by atomic force microscopy (AFM; Bruker, USA) and transmission electron microscopy (TEM; Hitachi Ltd., Japan; 2% phosphotungstic acid staining). Particle size and zeta potential measurements were conducted using a Zetasizer Nano ZS90 system (Malvern Panalytical Ltd., UK).

**Table 1:** Sequences of the ssDNA strands (5′→3′).

S1	ATTTATCACCCGCCATAGTAGACGTATCACCAGGCAGTTGAGACGAACATTCCTAAGTCTGAA
S2	ACATGCGAGGGTCCAATACCGACGATTACAGCTTGCTACACGATTCAGACTTAGGAATGTTCG
S3	ACTACTATGGCGGGTGATAAAACGTGTAGCAAGCTGTAATCGACGGGAAGAGCATGCCCATCC
S4	ACGGTATTGGACCCTCGCATGACTCAACTGCCTGGTGATACGAGGATGGGCATGCTCTTCCCG

### 4.2 Quantification of encapsulation efficiency and loading efficiency

The encapsulation efficiency (EE) and loading efficiency (LE) of tFNAs-TXA were determined using an ultrafiltration method combined with a ninhydrin-based colorimetric assay. Briefly, tFNAs and TXA were incubated at various molar feed ratios (ranging from 1:40 to 1:320) overnight at 4 °C to achieve stable physical adsorption. The unencapsulated free TXA was separated from the tFNAs-TXA complexes using centrifugal ultrafiltration filters (10 kDa MWCO, Amicon Ultra) by centrifugation. The concentration of free TXA in the filtrate was subsequently quantified by reacting it with ninhydrin reagent at 90 °C and measuring the absorbance at 570 nm using a NanoPhotometer. The EE and LE were then calculated based on the standard calibration curve of TXA.

### 4.3 In vitro drug release assay

The release kinetics of TXA from the tFNAs-TXA complex were evaluated using a dynamic dialysis diffusion technique. Briefly, the optimized tFNAs-TXA complexes (prepared at a 1:160 feed ratio) were sealed in dialysis bags (MWCO 3.5 kDa) and immersed in 50 mL of specific release media: PBS at pH 7.4 to mimic normal physiological conditions, and acetate buffer at pH 5.5 to mimic the mildly acidic intracellular endo/lysosomal microenvironment. The system was incubated at 37 °C under constant magnetic stirring to maintain sink conditions. At predetermined time intervals, 1.0 mL of the release medium was withdrawn for analysis and immediately replaced with an equal volume of pre-warmed fresh medium. The amount of released TXA was quantified using the ninhydrin colorimetric assay, and the cumulative release percentage was calculated over time.

### 4.4 Ex vivo skin penetration assay

The transdermal delivery capability of the nanostructures was visually evaluated using Cy5-labeled tFNAs. Briefly, excised dorsal skin tissues from C57BL/6J mice were mounted and treated topically with Cy5-tFNAs-TXA. After 24 h, the treated skin tissues were harvested, washed thoroughly with PBS to remove unpenetrated nanostructures on the surface, and embedded in optimal cutting temperature (OCT) compound for cryosectioning. Skin sections were counterstained with DAPI to visualize the cell nuclei. The fluorescence distribution and penetration depth of the nanocarriers across the skin layers were observed and imaged using a confocal laser scanning microscope (CLSM).

### 4.5 Cell culture and hyperpigmentation model

Murine melanoma B16 cells were acquired from Procell Life Science & Technology Co., Ltd. (Wuhan, China) and cultured in RPMI-1640 medium (Gibco, Thermo Fisher Scientific, Waltham, MA, USA) supplemented with 10% fetal bovine serum (FBS) and penicillin-streptomycin (Gibco, Thermo Fisher Scientific, Waltham, MA, USA) at 37 °C in a 5% CO_2_ humidified atmosphere. To simulate hyperpigmentation, cells were pre-stimulated with 1 μM α-melanocyte-stimulating hormone (α-MSH) (MedChemExpress, Monmouth Junction, NJ, USA) for 48 h. Subsequently, cells were treated for 24 h with the following regimens: (1) Control (α-MSH + Vehicle); (2) tFNAs (250 nM); (3) TXA (40 μM); and (4) tFNAs-TXA (250 nM tFNAs + 40 μM TXA). The concentrations were determined based on preliminary cytotoxicity screening.

### 4.6 Cell viability and proliferation assays

Cell viability was assessed using the Cell Counting Kit-8 (CCK-8). Cells were seeded in 96-well plates (1 x 10^4^ cells/well). Post-treatment, 10 μL of CCK-8 reagent (Sigma-Aldrich, St. Louis, MO, USA) was added and incubated for 2h at 37 °C. Absorbance was measured at 450 nm using a microplate reader (Tecan, Männedorf, Switzerland).

Cell proliferation was further evaluated using the Click-iT EdU Imaging Kit (Thermo Fisher Scientific, Massachusetts, USA). Cells were incubated with EdU for 2h, fixed with 4% paraformaldehyde (PFA), and permeabilized. Nuclei were counterstained with Hoechst 33342. Images were captured using a confocal laser scanning microscope (LSM 980; Carl Zeiss, Oberkochen, Germany), and the percentage of EdU-positive cells was analyzed using ImageJ software.

### 4.7 Measurement of melanin content

Cells (5 x 10^5^ cells/well) were harvested, washed with PBS, and lysed in 1 N NaOH at 80 °C for 60 min to solubilize melanin. The absorbance of the lysates was measured at 405 nm using a microplate reader (Tecan, Männedorf, Switzerland). The relative melanin content was normalized to the total protein concentration, determined by a BCA Protein Assay Kit (cat. no. PA115-01; Tiangen Biotech Co.; Ltd., Beijing, China).

### 4.8 Bioinformatics analysis

To predict the underlying mechanism, pharmacological targets of TXA were identified using the SwissTargetPrediction database (http://www.swisstargetprediction.ch), and melanogenesis-related targets were retrieved from the KEGG pathway database. Intersecting targets were subjected to pathway enrichment analysis using the clusterProfiler package in R. Significant pathways were filtered based on a p-value <0.05. To validate transcriptomic alterations, the GSE54359 dataset was obtained from the GEO database (https://www.ncbi.nlm.nih.gov/geo). This dataset reflects a dynamic "pigmentation oscillator" model, comprising 12 samples from murine and human skin systems. It tracks temporal transcriptomic changes across two consecutive cycles of pigmentation homeostasis to delineate the regulatory loops of melanogenesis. Data normalization and Principal Component Analysis (PCA) were conducted to evaluate sample distribution. Differentially expressed genes (DEGs) were identified using the limma package. Results were visualized via volcano plots (ggplot2) and heatmaps (ComplexHeatmap). Finally, a Sankey-Bubble plot was generated using ggalluvial to map DEGs to enriched pathways and illustrate the regulatory network.

### 4.9 Immunofluorescence staining

To assess the expression levels of melanogenic proteins, cells were fixed with 4% PFA, permeabilized using 0.1% Triton X-100, and blocked with 1% BSA for 1 h at room temperature. Subsequently, samples were incubated overnight at 4 °C with primary antibodies specific for Tyrosinase (Cat No. 31291-1-AP) and MITF (Cat No. 13092-1-AP), both purchased from Proteintech (Wuhan, China). Following washing, cells were probed with fluorophore-conjugated secondary antibodies (Cat No. SA00001-1; Proteintech) for 1 h. Nuclei were counterstained with DAPI. Images were acquired using a Carl Zeiss confocal microscope, and fluorescence intensity was analyzed via ImageJ.

### 4.10 qRT-PCR and western blot analysis

Total RNA was extracted using TransZol Up (TransGen Biotech, Beijing, China), and qPCR was performed using the TransStart® Top Green qPCR SuperMix (TransGen Biotech) with primers synthesized by Sangon Biotech (Shanghai, China). For Western blotting, proteins were separated by SDS-PAGE and transferred to PVDF membranes. Membranes were incubated overnight at 4 °C with primary antibodies against TYR (Cat No. 31291-1-AP), MITF (Cat No. 13092-1-AP), CREB (Cat No. 67927-1-Ig), and p-CREB (Cat No. 28792-1-AP) (all from Proteintech, Wuhan, China). Subsequently, HRP-conjugated secondary antibodies (Cat No. SA00001-0; Proteintech) were applied. Protein bands were visualized and quantified using Image J.

### 4.11 Animal experiments and UVB-induced pigmentation model

Female C57BL/6J mice (6 weeks old) were purchased from GemPharmatech Co., Ltd (Nanjing, China). All animal procedures were approved by the Institutional Animal Care and Use Committee (IACUC) of Sichuan Provincial People's Hospital (Approval No. LS-2025-428). Mice were anesthetized with 1% pentobarbital sodium. The dorsal skin was shaved and irradiated with 311 nm UVB light at a distance of 15 cm for 15 min daily. The UVB irradiance at the skin surface was measured to be 200 μW/cm^2^, which corresponds to a daily delivered dose of 180 mJ/cm^2^. The irradiation protocol consisted of 4 cycles (5 days of exposure followed by 2 days of rest), resulting in a total cumulative UVB dose of 3.6 J/cm^2^ over the 20 exposure days. Topical formulations were prepared by incorporating the active agents into an emulsion-based gel system. The vehicle consisted of a 1.8% (v/v) cold-process polymeric thickening emulsifier (Sepigel 305, comprising polyacrylamide, C13-14 isoparaffin, and laureth-7) uniformly dispersed and swollen in deionized water. The final applied concentrations of the active ingredients in the topical formulations were designed to match the in vitro studies (250 nM for tFNAs and 40 μM for TXA). Specifically, the formulations were prepared using a 5-fold dilution method: a 5× concentrated active stock solution (2 mL) was mixed with 0.18 mL of the liquid polymeric emulsifier, and deionized water was subsequently added to make up a final total volume of 10 mL. Following modeling, mice were randomly divided into 6 groups (n = 6) and treated topically with 2 mL of the respective formulations daily for 15 days. The animal grouping and topical treatment regimens were as follows: (1) Blank (No UVB); (2) Control (UVB + Vehicle); (3) tFNAs (250 nM); (4) TXA (40 μM); (5) tFNAs-TXA (250 nM tFNAs + 40 μM TXA); (6) Hydroquinone (HQ, 4% w/v).

### 4.12 Assessment of skin pigmentation

Photographs of the dorsal skin were taken on days 0, 5, 10, and 15. The degree of pigmentation was quantified using ImageJ software. A fixed region of interest (2 cm × 4 cm) was selected. Images were converted to 8-bit grayscale, and the area of hyperpigmentation (black spots) was calculated by subtracting the non-pigmented area from the total ROI area.

### 4.13 Histological analysis

Mice were euthanized after 15 days of treatment. Dorsal skin tissues were fixed, embedded in paraffin, and sectioned (4-5 μm). Masson-Fontana staining was performed to visualize melanin content. Masson's Trichrome staining was used to assess collagen density. Hematoxylin and Eosin (H&E) staining was conducted to measure epidermal thickness. Histological images were acquired using an inverted microscope and analyzed quantitatively using ImageJ.

### 4.14 Statistical analysis

Statistical analysis was performed using GraphPad Prism 9. Data are presented as the means ± SD. Significant differences were evaluated by one-way ANOVA followed by Tukey's post-hoc test for multiple comparisons. The exact number of independent biological replicates (n) is explicitly stated in the respective figure legends. P < 0.05 was considered statistically significant.

## CRediT authorship contribution statement

**Tao Cai:** Formal analysis, Investigation, Writing – original draft. **Junyang Huang:** Data curation, Software, Writing – original draft. **Li Chen:** Data curation, Formal analysis, Investigation, Methodology. **Yi Lin:** Methodology, Software, Visualization. **Kaiqun Liu:** Investigation, Methodology, Software. **Yuanyuan Deng:** Data curation, Methodology. **Jiajin Feng:** Data curation. **You Wang:** Conceptualization, Supervision, Writing – review & editing. **Xiaoyan Ding:** Conceptualization, Funding acquisition, Writing – review & editing.

## Declaration of competing interest

The authors declare that they have no known competing financial interests or personal relationships that could have appeared to influence the work reported in this paper.

## Data Availability

Data will be made available on request.
